# A Review of Ethnicity, Culture, and Acculturation Among Asian Caregivers of Older Adults (2000-2012)

**DOI:** 10.1177/2158244014566365

**Published:** 2015-02

**Authors:** Christina E. Miyawaki

**Affiliations:** 1 University of Washington, Seattle, Washington, USA

**Keywords:** Asian caregivers, acculturation, culturally sensitive, generations, immigration

## Abstract

This review identified domains of care experiences among studies of Chinese, Filipino, Japanese, Korean, and Vietnamese caregivers in the United States and Canada between 2000 and 2012. Using a narrative approach, 46 peer-reviewed journal articles were found through electronic databases and references. Considering caregivers’ assimilation to host countries, attention was given to their culture, socioeconomic resources, immigrant status, filial responsibility, generation, and acculturation. Three primary domains were identified across subgroups. The caregivers’ experiences domain was a strong sense of filial responsibility and its varied effects on caregiving experience; in the cultural values domain, reciprocity, and familism. In the acculturation domain, caregivers’ generations influenced their experiences. Because our society is rapidly changing demographically and culturally, studies of older adults and their caregivers that are not only inclusive of all racial/ethnic groups but also sensitive to specific racial/ethnic and cultural subgroup differences are necessary to inform policy and practice.

## Introduction/Background

The rapid increase of the U.S. aging population in general, as well as the growth of older populations of color are well documented (Administration on Aging, 2012). The increasing racial and ethnic diversity of the older population along with the dramatic growth of family caregivers in the United States raises important policy questions about whether supports for caregivers of older adults are adequate and appropriate within diverse communities, including immigrants. A step toward addressing this policy question is to better understand whether and how such supports vary across racial and ethnic groups. Moreover, variations by generation and acculturation are likely to influence immigrants’ caregiving experiences.

Previous researchers have conducted caregiving literature reviews with a focus on race, ethnicity, and culture. Articles on Black and some Hispanic caregivers were typically reviewed, with only few Asian caregiving studies included (Table 1). More recent reviews have included more samples of Asian caregivers, but focused on either one ethnic subgroup of Asian or a comparison of two cultures (Table 2). This literature review takes account of the increasing diversity among Asians. It synthesizes what has been found among studies of Asian caregiving in the United States and Canada including a sense of filial responsibility—one of the core cultural traditions among many Asian countries (Lai, 2010). It includes caregiver samples from the five largest Asian ethnic subgroups: Chinese, Filipino, Japanese, Korean, and Vietnamese. These ethnic subgroups comprise 12.1 million people, which is 70.4% of total Asian populations and 3.9% of the total U.S. population (Hoeffel, Rastogi, Kim, & Shahid, 2012). It also examines the similarities and differences in their caregiving experience in the United States or Canada. As immigrants, Asian caregivers may be disposed to cultural or behavioral assimilation. Thus, special attention was paid to caregivers’ culture, socioeconomic resources, immigrant status, sense of filial responsibility, immigrant generation, and acculturation across these ethnic subgroups.

## Methods

### Scope of Review

This analysis used a narrative approach similar to work done by Dilworth-Anderson, Williams, and Gibson (2002). This present study focused specifically on Asian caregiving experiences described in 46 articles published in peer-reviewed U.S. and Canadian journals between 2000 and 2012. A narrative literature review was appropriate because it can not only compile a vast scattered range of articles on a particular topic (i.e., caregiving of older adults by five ethnic subgroups of Asian immigrant family caregivers) but also grasp larger abstract research questions, explore underlying meanings, and link them to see similarities and differences.

### Search Strategy

Articles were selected based on their focus on informal/family caregiving relationships between caregivers (child/children) and dependent older adults (parent(s), relative(s), and friend(s), etc.). The majority of articles were descriptive reports of one ethnic subgroup, but some included several different Asian ethnic subgroups or different racial groups (i.e., White, Black, and Asian). In the latter case, only reports of Asian subgroups were included in this review. Reports on professional caregivers in long-term care facilities, as well as those caregivers who live outside of North America were excluded from the selection.

Several databases such as Academic Search Complete (EBSCO), CINAHL Plus, ERIC, Medline, PsycINFO, and PubMed were first used to find and select articles that meet the above criteria. Terms such as Asian caregivers, informal care-giving, culture, dementia, elderly, filial piety, filial responsibility, ethnicity, immigrant, refugees, Chinese American, Chinese Canadian, Japanese American, Japanese Canadian, Korean American, Filipino American, and Vietnamese American were searched within the titles and abstracts. References from selected articles were also used to identify additional studies.

### Data Organization

After selecting the articles for inclusion, an information sheet was first created for each article, which summarized the theory used, sample, measures, research design, and key findings. Second, separate tables were constructed for Chinese, Filipino, Japanese, Korean, and Vietnamese care-givers, and the articles were organized by focus of research, research design, sample characteristics, and key findings. Theory was not included as a category because few articles utilized a theoretical or conceptual framework. Third, research topical domains, defined as “common areas of research that comprise a broad framework” (Dilworth-Anderson et al., 2002, p. 238), were created covering broad caregiving issues experienced by Asian caregivers. These domains were based on the most commonly discussed topics, which were brought up most frequently across ethnic subgroups or strongly expressed among a few subgroups. The three research topical domains are (a) caregivers’ experiences, (b) cultural values, and (c) acculturation, that is, adjustments and adaptations to the dominant majority by minorities. Foci are nested within these three domains. Table 3 shows research topical domains, foci, and topics divided by ethnic subgroups and numbers of articles in each category. Tables 4 to 8 present corresponding article numbers and their characteristics sorted by research focus, design, sample, and key findings.

## Research Design and Sample Characteristics

Because the majority of studies were exploratory in nature and due to challenges of recruiting samples of Asian caregivers, most used nonprobability sampling (40 out of 46) typically through advertising in ethnic-specific senior and community centers, newspapers, radios, and using snowball sampling. Random sampling studies were limited to Lai's (2007, 2009a, 2009b, 2010) and Lai and Thomson's (2009) 339 Chinese Canadian and Casado and Sacco's (2012) 146 Korean American caregivers only. Four types of data collection techniques were used: face-to-face interview, phone survey/interview, focus group, and questionnaire/mail survey. Some studies utilized the same samples, were written by the same author(s), and produced separate articles (Jones, Jaceldo, Lee, Zhang, & Meleis, 2001; Jones, Zhang, Jaceldo-Siegl, & Meleis, 2002; Jones, Zhang, & Meleis, 2003; Lai, 2007, 2009a, 2009b, 2010; Lai & Thomson, 2009). The majority of caregivers tended to be women regardless of ethnic subgroups. All care-givers except Japanese used their preferred languages in their written formats and interviews. This language choice is closely related to the sample characteristics of caregivers, their immigrant status and generations. Caregivers in all the studies except the two Chinese Canadian studies (Chappell & Funk, 2011; Funk, Chappell, & Liu, 2011), and in the studies of Japanese caregivers are first-generation immigrants, and thus, they speak their native languages and carry their native cultures. In contrast, Japanese caregiver samples are second- and third-generation immigrants, and therefore, their native language is English.

## Research Domains

**Figure 1:**
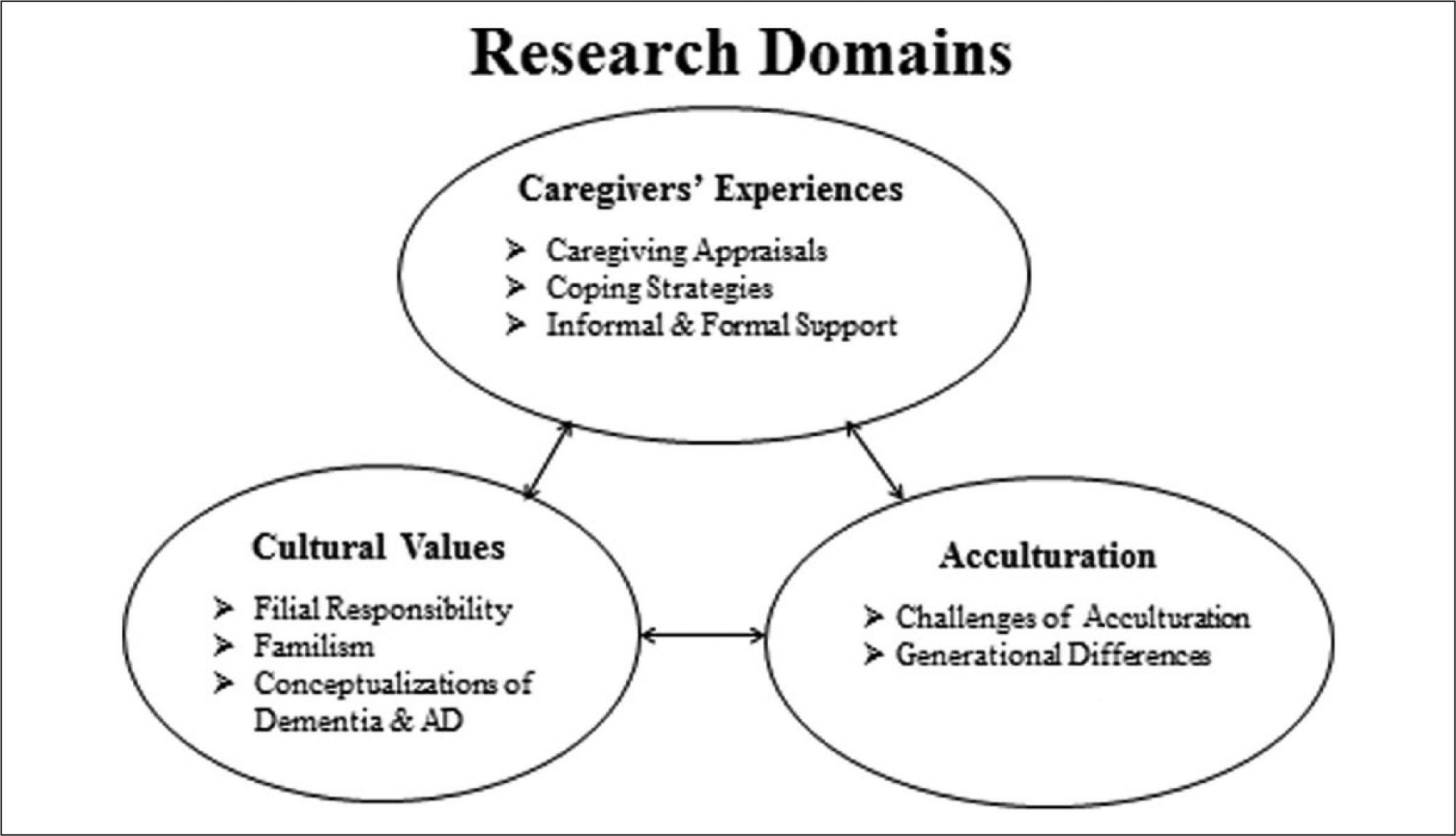
Diagram of research domains and dimensions. *Note*. AD = Alzheimer's disease.

Figure 1 shows a diagram of the domains and dimensions of this review.

### Domain 1: Caregivers’ Experiences

Thirty-six unduplicated articles addressed issues of caregiving appraisal (19 articles), coping strategies (16 articles), and informal and formal support (23 articles) in relation to Asian immigrant caregivers’ experiences.

#### Caregiving appraisal

In their definition of caregiver appraisal, Lawton, Kleban, Moss, Rovine, and Glicksman (1989) included positive, neutral, and negative aspects of “caregiving stress” (p. P61). Their definition recognizes that a stressor for some people may not be a stressor for others, especially in relation to the caregivers’ culture (Lai, 2010). In contrast, these 36 articles were limited to positive and negative appraisals only. Examples of positive appraisals included Chinese Canadian caregivers’ caregiving experience as an expected stage in their lives (Ho, Friedland, Rappolt, & Noh, 2003), rejection of caregiving as a burden (Spitzer, Neufeld, Harrison, Hughes, & Stewart, 2003), and strong identity with filial responsibility and better health (Lai, 2009a, 2009b, 2010); Chinese Americans expressed caregiving as a cultural obligation (Tang, 2011), strong belief in filial responsibility (Holland, Thompson, Tzuang, & Gallagher-Thompson, 2010), psychological reward of caring (Zhan, 2004), and role integration/satisfaction and physical health and personal growth (Jones et al., 2001; Jones et al., 2002; Jones et al., 2003). Korean Americans’ strong sense of filial obligation (Kim & Theis, 2000) and social support (Lee & Bronstein, 2010) were the major factors in their positive caregiving experience. Vietnamese American caregivers accounted for their prayers as a means of their strength and motivation for caregiving (Hinton, Tran, Tran, & Hinton, 2008).

For negative appraisals, Filipino American (Jones et al., 2001) and Japanese American caregivers (Anngela-Cole & Hilton, 2009) reported high levels of mental stress, despite their acceptance of caregiving. However, Japanese American caregivers who valued their caregiving experienced lower levels of depression and greater life satisfaction than those who thought of caregiving as a burden (Anngela-Cole & Hilton, 2009). As Korean Americans strongly believe in fulfilling their filial duty as life satisfying, they expressed being caregivers as their “privilege.” But they also implied being caregivers with a negative connotation because there is no other available person or no other choice but becoming a caregiver (Kim & Theis, 2000). Vietnamese caregivers were confused by the nature of dementia and Alzheimer's disease (AD) and thought of mental illness attributed by haunted spirit (Hinton, Tran, Tran, & Hinton, 2008).

#### Coping strategies

Coping strategies are one of the most frequently mentioned aspects (*n* = 16) of the dimensions of caregiving domain. The vast majority of articles reported a strong belief in filial responsibility, spirituality, religion, and prayers, and informal network support as the three major coping strategies.

Caregivers’ strong belief in filial responsibility was the most prevalent coping strategy used across all ethnic sub-groups. A sense of filial values and cultural commitment to caring for aging parents (Funk et al., 2011; Jones et al., 2003; Lai, 2010), family loyalty and responsibility, and respect for elders (Spitzer et al., 2003) are coping techniques for Chinese American and Chinese Canadian caregivers. Simply accepting their caregiver role to fulfill their filial duty (Ho et al., 2003) and Chinese values of commitment to “hard work,” “self-improvement,” and a sense of “emotional hardiness” (Holland et al., 2010, p. 122) were other ways to handle difficult caregiving situations. Determination to care at all costs and personal sacrifice (Jones et al., 2002; Jones et al., 2003) was Filipino American caregivers’ coping style.

All the Vietnamese American caregivers in this review were either Catholic or Buddhist and they often used concepts such as karma, blessings, grace, and peace of mind to express spiritual dimensions of their caregiving experiences (Liu, Hinton, Tran, Hinton, & Barker, 2008). Chinese and Filipino American caregivers considered caring for aging parents as their highest calling. Through their religious faith, they gained strength, developed meaning of their caregiving experience, managed their caregiving responsibility, and grew stronger as a person (Jones et al., 2003). Other Chinese American caregivers reported their religion, meditation, and prayers as a source of comfort, and their spiritual beliefs gave them strength to cope and continue to be good caregivers (Vickrey et al., 2007).

Chinese Canadian (Funk et al., 2011; Ho et al., 2003) and Chinese American (Jones et al., 2002; Jones et al., 2003) caregivers used only support within their family members as a coping strategy, because caregiving responsibilities cannot be transferred to outsiders and caregiving is assumed or considered as a women's role (Spitzer et al., 2003). Having a large family support rather than a network of friends alleviated Korean Americans’ caregiver burden (Casado & Sacco, 2012; Han, Choi, Kim, Lee, & Kim, 2008; Lee & Bronstein, 2010; Yong & McCallion, 2003). Because the size of family networks may be smaller than those in their home country, Filipino American caregivers mobilized other family resources such as their spouses and siblings as much as possible (Jones et al., 2003). The situation of Japanese American caregivers is not as clear. Although third-generation Japanese American caregivers were found to spend less caregiving hours and displayed a less positive attitude to caregiving compared with Caucasian caregivers (Anngela-Cole & Hilton, 2009), other Japanese Americans depended on their network support rather than utilizing formal services as their coping strategies.

#### Informal and formal support

The issue of informal and formal support use is another major aspect of caregivers’ experiences. This is of practical importance due to caregivers’ immigrant status as first-generation immigrants who experience linguistic barriers to service use and carry cultural values of their homelands. Chinese American, Chinese Canadian, Korean American, and Vietnamese American caregivers expressed language as a barrier to use formal services (Han et al., 2008; Kong, Deatrick, & Evans, 2010; Strumphf, Glicksman, Goldberg-Glen, Fox, & Logue, 2001; Vickrey et al., 2007; Zhan, 2004). Other barriers mentioned by these caregivers included structural barriers such as a lack of appropriate formal services in terms of language (Jones et al., 2003; Zhan, 2004), culturally sensitive services (Han et al., 2008; Kim, 2009; Spitzer et al., 2003; Tang, 2011; Zhan, 2004), and services for refugees (Strumphf et al., 2001).

Chinese, Korean, and Vietnamese caregivers felt a lack of emotional support (Ho et al., 2003; Kim & Theis, 2000; Levy, Hillygus, Lui, & Levkoff, 2000; Zhan, 2004), as well as financial and material support (Kim & Knight, 2008; Lai & Thomson, 2009) from their families and ethnic communities. In addition, caregivers themselves tended not to use formal services due to their cultural beliefs and/or cultural taboos of using formal, professional services (Han et al., 2008; Jones et al., 2002; Jones et al., 2003; Kong et al., 2010; Lai, 2007, 2010; Levy et al., 2000; Spitzer et al., 2003; Strumphf et al., 2001; Zhan, 2004). Despite their first-generation immigrant status, more educated, wealthier Chinese caregivers employed Chinese-speaking paid-caregivers for their parents as if they are kin members (Lan, 2002). Filipino American caregivers showed mixed feelings about the use of formal services. Despite their filial commitment, due to their immigration and economic realities in a new country, they were open to using formal services, even though there were concerns about the care recipients’ shame (Kimura & Browne, 2009). Contrary to other Asian caregivers, Japanese American caregivers and their family members accepted the use of formal services. Their core philosophy of “sharing” of caregiving includes caregiving by both family and formal services, as long as these services meet care recipients’ needs, are offered by high quality staff, provide recipients with privacy and a sense of home, and are culturally congruent. With these requirements, they considered formal services as an extension of family caregiving (Young, McCormick, & Vitaliano, 2002a, 2002b).

In summary, all five subethnic groups of caregivers tended to use informal rather than formal support typically by counting on their family members. This was found particularly among first-generation caregivers, probably because of their linguistic and cultural barriers. However, a more acculturated subethnic group of caregivers (e.g., Japanese) and those who could afford to hire paid-caregivers (e.g., more educated Chinese families) were more open to the idea of utilizing outside formal help.

### Domain 2: Cultural Values

The cultural values domain was identified in 36 articles that addressed filial responsibility (25 articles), familism (13 articles), and conceptualization of dementia and AD (7 articles).

#### Filial responsibility

Filial piety is a fundamental Confucian values common among many Asian cultures and historically teaches respect for parents, emphasizes on intergenerational relationships, and puts family over individual interests (Sung, 2001). In Asian countries and cultures, adult children are traditionally expected to sacrifice their physical, financial, and social needs for the benefits of their aging parents. They take a family-centered approach to fulfill their filial responsibility (Dai & Dimond, 1998) in contrast to an individualistic approach characteristic of Western cultures (Chappell & Funk, 2011).

As this sense of filial responsibility has been embedded into Asian culture and continues to have a strong impact on people's lives and parent–child relationships (Lai, 2010), in this review, the subject of filial responsibility emerges under more than one domain. Chinese Canadian caregivers recognized the differences in filial attitudes between White-Canadian caregivers (i.e., Western culture) and themselves (i.e., Chinese culture): Caucasian Canadian caregivers showed a lower sense of filial responsibility and provided less financial assistance to their parents compared with Chinese Canadian counterparts (Chappell & Funk, 2011; Funk et al., 2011; Ho et al., 2003): The stronger their filial commitment, the more positive their caregiving experiences (Lai, 2007, 2010). They identified filial responsibility as Asian or Chinese cultural values (Holland et al., 2010) and emphasized collectivity and Confucian ideals (Tang, 2011). Chinese American caregivers also expressed their conventional lifelong reciprocal obligation for parental care (Hsueh, Hu, & Clarke-Ekong, 2008) and performed “transplanted filial values” (Jones et al., 2002, p. 204) due to their immigrant status. They call it “transplanted” because their deep commitment and cultural values of filial responsibility were developed before immigrating to the United States; however, their filial practice was implemented in the United States. Similar Asian filial values in a “translated,” but mutually agreed form within the family were found among later generations of Japanese Canadian families (Kobayashi, 2000; Kobayashi & Funk, 2010). While they recognized their changing filial expectations and duties across generations due to their immigration to the United States (Kim, 2009), Korean American caregivers tried to maintain the “Korean way of thinking”: family and filial responsibility as “a fundamental cultural belief of caregiving” (Kong et al., 2010, p. 322). Vietnamese American caregivers seemed to be over-whelmed with their new lives in the United States, but strongly endorsed a sense of filial responsibility and care for their older parent(s)/relatives (Strumphf et al., 2001; Yeo, Tran, Hikoyeda, & Hinton, 2001).

Due to their immigrant status and consequential financial necessity, caregivers are forced to play multiple roles, not only as a caregiver for elders and other family members but also as an employee (Jones et al., 2002; Kim & Theis, 2000; Lai, 2007; Lee & Farran, 2004; Spitzer et al., 2003). In contrast to first-generation Asian immigrants, Japanese caregivers are the second and third generations and, therefore, are more acculturated to Western cultural values. In addition, we need to acknowledge that Japanese Americans faced oppression from their World War II internment experience and aftermath. These situations made their acculturation a requirement for survival. Despite their acculturation, both second- and third-generation Japanese Canadian caregivers seemed to be congruent in both in degree (strong) and content (important) of their sense of filial obligation (Kobayashi, 2000; Kobayashi & Funk, 2010). Working outside the home is a norm for Japanese Canadian caregivers, and more family participation and sharing of caregiving duties seemed to be expected (Kobayashi, 2000; Young et al., 2002a, 2002b).

#### Familism

Familism emphasizes “the family over the individual, showing respect for elders, and honoring the family name” (Schwartz, 2007, p. 102), and it is often contextualized within “family-centered cultural traditions and interpersonal impacts of providing care” (Scharlach et al., 2006, p. 139). It is different from a sense of filial responsibility, which is based on the individual, while familism is a group or collectivist value. Studies that identify familism as a cultural value posit that it promotes respect for elders within the family. Moreover, sharing of caregiving responsibility as a family unit may serve as a protective factor for caregivers’ mental health (Knight et al., 2002; Scharlach et al., 2006). Korean American caregivers most frequently mentioned familism, but in negative ways such as an association between strong familism and high levels of caregiving burden and distress, and caregivers’ poorer health (Knight et al., 2002). Yong and McCallion's (2003) study discussed hierarchical and unjust relationships within their family members causing Korean American female caregivers *hwabyung*, diagnosed as somatization disorder, depression, and anxiety often caused by feelings of oppression (Park, 2004).

Respect for elders was strongly voiced by Chinese American (Hsueh et al., 2008; Jones et al., 2002; Spitzer et al., 2003) and Filipina American caregivers (Jones et al., 2002), despite parent(s)’ symptoms of dementia (Liu et al., 2008). Filipina caregivers’ reciprocal attitude formed a strong bond between the elder's guardianship, protection and kindness, and caregivers’ caregiving services (Kimura & Browne, 2009). Korean American female caregivers respect their parent(s) and in-law(s) as something that was expected as daughters and daughters-in-law within a family unit, and gain a sense of fulfillment in caregiving (Kim & Theis, 2000).

Due to their immigrant status and smaller network sizes, sharing caregiving responsibility expanded to adult sons and husbands (Jones et al., 2002) and children (Kobayashi, 2000). Moreover, as mentioned earlier, Japanese American and some Chinese American families hired bilingual Japanese or Chinese paid-caregivers as their fictive kin (Hsueh et al., 2008; Lan, 2002; Young et al., 2002a). They maintain a core concept of familism but use a modified approach to filial responsibilities.

#### Conceptualizations of dementia and AD

Some factors among Chinese and Vietnamese caregivers seem to prevent them from seeking services for their elders raised issues related to dementia/AD. They believe that all elders become forgetful and confused as they age, and hence arranging for medical attention to address these symptoms is not their priority (Yeo et al., 2001); this contrasts with White caregivers who are more likely to consider dementia as a medical condition that needs to be addressed (Vickrey et al., 2007). Although they consider having symptoms of dementia as part of a normal aging process, a strong social stigma is attached to its symptoms, and in turn, hinders caregivers from seeking professional medical help (Gray, Jimenez, Cucciare, Tong, & Gallagher-Thompson, 2009; Levy et al., 2000; Yeo et al., 2001). Others believe that causes of dementia are due to care recipients’ mental illness, personality problems, or substance abuse, and therefore, bring shame to the family (Liu et al., 2008) and a fear of contagion (i.e., discouraging marriage into a family with a history of mental illness) (Yeo et al., 2001). Some Vietnamese caregivers connect their misfortune of having their loved ones with dementia/AD to religion and seek spiritual explanations. They consider the causes of loved ones’ illnesses as “the manifestation of God's will” or “curses or spiritual possessions” (Hinton et al., 2008, p. 10). Concerned with what other people in the community think about the diagnosis of dementia (Vickrey et al., 2007), they keep the diagnosis of dementia/AD within the family; they exclude relatives with dementia from social interactions within their ethnic community (Liu et al., 2008; Zhan, 2004), whereas White caregivers do not consider dementia as something to hide (Vickrey et al., 2007). Translated words of AD and/or dementia imply the meaning of “stupidity” (Vickrey et al., 2007) and/or “crazy” (Yeo et al., 2001), and thus, a diagnosis of dementia brings a social stigma. In addition, acknowledging that they have a problem in the family to other community members is not a norm in Chinese culture (Vickrey et al., 2007). These physiological, psychosocial, and spiritual/religious concepts of dementia and AD have posed as additional attributing factors in Chinese and Vietnamese caregivers’ care challenges.

### Domain 3: Acculturation

The acculturation domain includes 20 unduplicated articles. These discussed various challenges borne out of immigration to the United States/Canada covering challenges of acculturation (19 articles) and generational differences of beliefs in filial responsibility due to acculturation (8 articles).

#### Challenges of acculturation

Because all except Japanese American and Japanese Canadian caregivers are first-generation immigrants, they faced challenges of assimilation to their new homelands. If these caregivers brought younger family members with them to raise while caring for their older parents, their challenges were compounded by the needs of three different generations. All the caregivers in the studies reviewed had gone through some processes of assimilation and faced challenges of maintaining their traditional cultural beliefs. Chinese Canadian caregivers tried to assimilate into Western culture while retaining strong Chinese cultural values (Ho et al., 2003); however, Lan (2002) reported that traditional Chinese cultural norms of filial responsibility and parental authority were modified after families resettled in the U.S. Chinese and Filipino American caregivers experienced a sense of being in transition because of adjustment to new roles and changes in their beliefs, values, and priorities from Asian to Western values (Jones et al., 2002). These challenges were exacerbated by an ongoing process of learning a new language, new social standards, and functioning in a new environment (Jones et al., 2003). Korean American caregivers reported conflicts among family members because different family members acculturated at different rates and have different values or beliefs in relation to caregiving (Han et al., 2008; Lee & Bronstein, 2010). Others experienced acculturation stress and new social roles as employees (Lee & Farran, 2004) or as caregivers to their own parent(s), which would have been the roles of daughters-in-law only if they had remained in Korea (Chun, Knight, & Youn, 2007). Vietnamese American caregivers’ challenges were primarily acquisition of English language and assimilation to new Western culture and American lifestyle. The most acculturated Japanese American and Japanese Canadian caregivers reported higher burden or depression if they held stronger Asian cultural values (Knight et al., 2002). Thus, their care-giving style has been modified to a family–community style, a combination of family and paid outside resources (Kobayashi & Funk, 2010; Young et al., 2002a). Challenges faced by all Asian caregivers in this study reflect both the recency and immigrant generations of caregivers.

In terms of challenges of immigrant status, Chinese American caregivers expressed the hardship of emigrating at an older age (Levy et al., 2000), and Chinese Canadian care-givers reported that caregiving is costly in Canada due to their smaller support network and their low-wage jobs (Spitzer et al., 2003). Korean American caregivers wondered if their aged-parent(s) might have been better taken care of if they stayed in Korea. Moreover, caregivers themselves felt lonely due to a lack of emotional support, and wondered whether their caregiving experiences would have been different in Korea instead of the United States (Kim, 2009).

#### Generational differences of beliefs in filial responsibility

Chinese, Filipino, and Korean immigrant caregivers uniformly voiced the conflicts in terms of filial responsibilities and expectations between the generations of caregivers and their aging parent(s) (Ho et al., 2003; Jones et al., 2002; Kim, 2009) or among the three generations—caregivers’ parent(s), caregivers, and caregivers’ children (Han et al., 2008; Jones et al., 2003). As to Japanese American and Japanese Canadian caregivers, their patterns varied, as mentioned under familism. Young and colleagues (2002b) concluded that generational perceptions become more complex and diverse as the Japanese American generations distance themselves from the first-generation immigrants.

## Summary

Forty-six peer-reviewed articles on Chinese, Filipino, Japanese, Korean, and Vietnamese American and Canadian family caregivers of older adults from 2000 to 2012 were compared and analyzed in terms of the domains of the care-givers’ experiences, cultural values, and acculturation. Regardless of ethnic subgroups and their immigrant generations, all caregivers expressed their deep commitment to caring for their loved ones and a strong sense of filial responsibility. However, depending on the immigrant generations of caregivers, their needs and approaches to caregiving differed, which may be a reflection of acculturation to the Western way of caregiving.

This review makes several contributions to the existing knowledge base on caregiving. To the author's knowledge, this is the first study, which has examined multiple ethnic subgroups within Asian family caregivers. Second, in terms of filial responsibility, a strong sense of filial responsibility was found and had positive effects across the five ethnic subgroups. However, Japanese American and Japanese Canadian caregivers redefined filial responsibility to include utilization of formal services that they viewed as professionally and culturally appropriate. This trend reflects immigrant generational differences and acculturation levels. Because Japanese caregivers are second and third generations, they do not have a language barrier and are culturally more receptive to Western norms of caregiving, which include using formal resources. In addition, recency of immigration to the host countries affects caregivers’ financial resources and their caregiving experiences. Due to first-generation immigrant caregivers’ language barriers, they may not be able to obtain well-paid positions, and thus a lack of resources makes their caregiving more challenging.

In this literature, the Asian subgroup is divided and characterized by generation in the host country. All Filipino, Korean, and Vietnamese caregiver samples were exclusively first-generation immigrant caregivers. A few Chinese Canadian studies (Chappell & Funk, 2011; Funk et al., 2011; Lai, 2007, 2009a, 2009b, 2010; Lai & Thomson, 2009) included second-generation caregivers. Japanese American and Japanese Canadian samples used a combination of second and third generations (Knight et al., 2002; Kobayashi & Funk, 2010; Young et al., 2002a, 2002b), or third generation only (Anngela-Cole & Hilton, 2009). These sample characteristics reflect the history and the length of residence of each ethnic subgroup in the United States or Canada. As the age groups of caregivers are within the same range (i.e., middle-age adult children), the differences of caregiver generations appear to influence their experiences across different ethnic subgroups of Asians.

The primary foci of the articles were divided into three main domains: caregivers’ experiences (36 articles), cultural values (36 articles), and acculturation (20 articles). In the caregivers’ experiences domain, positive appraisals of care-giving despite its hardships, caregivers’ strong beliefs in filial responsibility, and frequent use of informal support within their family members were commonly seen across these groups. Barriers to formal service use as well as lack of appropriate services, primarily due to language difficulties and cultural differences, were found among Chinese, Korean, and Vietnamese caregivers. In contrast, an openness to accept formal services occurred among more acculturated Japanese and some Chinese American caregivers. The availability of funds to hire outside help may be an important factor to consider in addition to cultural factors.

In terms of the cultural values domain, intense feelings of filial responsibility and reciprocity and familism were reported across all ethnic subgroups. As a result of their strong filial responsibility, Chinese, Filipino, and Korean caregivers expressed a dilemma of not being able to provide as much care as they wished. At the same time, they strongly endorsed caregiving as their reciprocal obligation for their parents’ past services. Second-generation Japanese American caregivers’ lower familism scores compared with other Asian groups were likely due to their higher level of acculturation (Knight et al., 2002). Symptoms of dementia are viewed as part of a normal aging process, but with negative connotations among Chinese and Vietnamese caregivers (Gray et al., 2009; Liu et al., 2008; Yeo et al., 2001).

The acculturation domain reflects caregivers’ length of residency in their host counties and their acculturation levels. Because the majority of caregivers themselves emigrated as first generation and at the same time are caring for their first-generation aging parent(s) or relative(s), all caregivers experience conflicts between their traditional filial beliefs (i.e., Asian) and those of the host countries (i.e., Western). Moreover, their immigrant status and new environments have made their caregiving role more difficult because of a new language, employment responsibilities, and a smaller social support network. Although generational differences in regard to degree and content of filial responsibility among Japanese caregivers vary, clearer generational differences were found among other ethnic subgroups of immigrants.

## Study Limitations

This review has several limitations. Although an attempt was made to be inclusive of all databases and reference lists, some relevant articles and very recently published studies may not have been captured due to the specific criteria and strategies used. Because of the small set of U.S. studies, studies of Canadian caregivers were included, but this added another layer of complexity to this study. For example, demography, migration patterns, and health care systems differ between the United States and Canada, but these factors are difficult to tease out when examining caregivers’ experiences. Another limitation is that no studies were found on Filipino, Korean, and Vietnamese Canadian caregivers within the selected time period. The vast majority of studies used nonprobability sampling with small sample sizes. Any concluding claims or suggestions made in such studies should be treated with caution, not as “generalizable empirical statements” but rather “testable theoretical assertions” (Jiménez, 2004, p. 79). As the Asian caregiver populations are small to begin with, face-to-face interviews and focus groups, for instance, are appropriate data collection techniques; in addition, it is challenging to obtain a probability sample for some of subethnic groups (e.g., Vietnamese). However, as some studies have done, using a combination of both quantitative and qualitative methods may help improve the generalizability of the results. Last, this study was conducted as a narrative literature review. However, it could have been approached and presented as a systematic review of qualitative research studies with filial responsibility as an emerging theme among the five ethnic subgroups of Asian caregivers, because the vast majority of articles included in this review were based on qualitative research.

## Future Research

Based on this review, a number of suggestions for future studies can be made. As previous reviews noted (Dilworth-Anderson et al., 2002), a lack of a conceptual framework remains. In addition to the stress and coping model, theories/ models that are able to capture caregivers’ particular situations in long-term relations, such as life course perspective (Elder, 1998) and role integration theory (Meleis, Norbeck, Laffrey, Solomon, & Miller, 1989), could be incorporated into future studies. Furthermore, Asian immigrants come from diverse sociodemographic populations, and their living situations and circumstances reflect different relationships between caregivers’ home and host countries as well as various historical and contemporary settlement patterns in their new homes. Therefore, theoretical perspectives for future research need to be relevant to caregivers’ countries of origin and culture and use cross-culturally appropriate instruments/measurement, including culturally appropriate translated questionnaires (Kong, 2007; Sun, Ong, & Burnette, 2012).

Generational attitudinal differences toward filial responsibility, especially among later generations of caregivers, should be further explored. As some of the review articles revealed, different generations showed varied attitudes toward filial responsibility depending on the levels of acculturation. These attitudinal differences are influenced by the history and the political positionality between caregiver's native and the host countries and within the host country, a continuation of incoming immigrants from caregivers’ home countries, and other sociodemographic variables. First-generation immigrant caregivers will age whereas their second-generation children will grow up as Americans or Canadians. Eventually, second-generation children will face their caregiving phase, as has occurred among Chinese and Japanese immigrant families. Therefore, it would be beneficial to examine further the generational differences of the level of filial responsibility and resultant needs between current and later generations of Asian immigrant caregivers. It is assumed that language would not be a barrier to later generations of caregivers and that they would be more familiar with health care systems in the host country compared with the first-generation immigrant caregivers. Given these shifts across generations, a critical future question might be what are the culturally appropriate theoretical models and culturally sensitive instruments to use when studying later generations of Asian immigrant caregivers.

It is striking that there was no mention of gender of care-givers in all except Lai and Thomson's (2009) article. It was assumed that all the caregivers are women, which reflects Asian cultural expectations of caregivers (Jones et al., 2003; Lai, 2010). The reality of having a smaller social network size within the host country and holding outside employment by female caregivers has made filial caregiving more challenging not only for female caregivers but also for other family members. Each family has to modify their roles and male relatives and children may have to more actively participate in caregiving responsibilities. Thus, gender issues in caregiving responsibilities, such as what kind of care roles and how much involvement are expected and taken by female and male relatives should be investigated.

The increasing aging population of color and their care-givers (Sun et al., 2012) may be reflected in the growth of Asian caregiving studies in recent years. The U.S. Census Bureau projected that by 2043, non-Hispanic White population will no longer be the majority (West, Cole, Goodkind, & He, 2014). Asian American population will increase from 5.1% (2010) to 7.4%-9.7% (2050) (Day, 1996), and especially older Asians (65 years and older) will grow from 9.3% to 21.9% in the same period (Vincent & Velkoff, 2010). This may also affect the increase of diseases such as dementia and AD among older adults, and attention to racial and ethnic disparities in prevention and interventions. To support an increasingly diverse older population, it is important to pay attention to their caregivers’ physical and mental well-being and understand their situations at new host counties. As captured by this review, due to their immigrant status and recency, their filial caregiving challenges have been exacerbated by linguistic barriers, traditional cultural beliefs, and generational differences in acculturation levels.

Social service agencies, particularly ethnic-specific agencies with bilingual and bicultural social workers, should reach out and encourage ethnic elders and their caregivers to use a thorough biopsychosocial geriatric assessment to detect an early onset of dementia/AD. They should also provide educational programs on caregiving for immigrant caregivers in their native languages. In collaboration with other Asian social service sectors, annual city-wide Pan-Asian health fair, which consists of all volunteer health care providers, for example, can help providing the opportunity for these vulnerable populations and their caregivers to check their health status. Furthermore, the government can support these efforts by encouraging translators and translated versions of health care materials to be available at all social service sectors.

Because our society is rapidly changing demographically and culturally, ongoing studies of older adults and their care-givers that are not only inclusive of all racial and ethnic groups but also sensitive to specific racial, ethnic, and cultural group differences are necessary. It is an opportune time to conduct comprehensive rigorous studies focusing on different ethnic subgroups of Asians using both quantitative (e.g., population based) and qualitative (e.g., in-depth face-to-face interviews) methods that can lead to culturally sensitive practices for older adults and their caregivers.

## Figures and Tables

**Table 1:** Literature Reviews Focused on Race, Ethnicity, and Culture.

Authors (publication year)	Periods reviewed	Number of articles	Number of Asian caregiver articles
Connell and Gibson (1997)	1985-1995	12	0
Dilworth-Anderson, Williams, and Gibson (2002)	1980-2000	59	3
Janevic and Connell (2001)	1996-2000	21	5

**Table 2:** Literature Reviews Focused on Asian Caregivers.

Authors (publication year)	Periods reviewed	Number of articles	Ethnic groups
Kong (2007)	1966-2005	32	Korean, Korean American, Caucasian American
Mokuau and Tomioka (2010)	1980-2007	22	Japanese, Japanese American
Napoles, Chadiha, Eversley, and Moreno-John (2010)	1980-2009	20	Chinese American or Asian
Sun, Ong, and Burnette (2012)	1990-2011	18	Chinese American

**Table 3:** Research Topical Domains of Caregiving Studies and Numbers by Ethnic Subgroups (2000-2012).

Domain	Topical focus	Chinese American/Canadian (Ref. No.) (*n* = 21)	Filipino American (Ref. No.) (*n* = 4)	Japanese American/Canadian (Ref. No.) (*n* = 6)	Korean American (Ref. No.) (*n* = 11)	Vietnamese American (Ref. No.) (*n* = 4)
Caregivers’ experiences	Caregiving appraisal					
	Positive appraisal	10^(4, 5, 7, 8, 11, 12, 13, 18, 19, 21)^			2^(35, 41)^	1^(43)^
	Negative appraisal	2^(6, 9)^	1^(22)^	1^(26)^	3^(32, 33, 35)^	1^(43)^
	Coping strategies					
	Spirituality/religion/prayers	2^(9, 20)^	1^(24)^			1^(43)^
	Strong belief in filial responsibility	6^(2, 4, 5, 9, 13, 18)^	2^(23, 24)^			
	Informal network support	5^(2, 4, 8, 9, 18)^	1^(24)^	1^(26)^	4^(32, 34, 41, 42)^	
	Informal and formal support					
	Lack of support	4^(4, 14, 16, 21)^			2^(35, 36)^	
	Barriers to use of formal services	2^(20, 21)^			2^(34, 39)^	1^(45)^
	Lack of appropriate formal services	4^(9, 18, 19, 21)^			1^(34, 37)^	1^(45)^
	Less use of formal services	7^(8, 9, 10, 13, 16, 18, 21)^			1^(34, 39)^	1^(45)^
	Open to use of formal services	5^(2, 4, 6, 15, 19)^	1^(25)^	2^(30, 31)^		
	No. of articles	18	4	3	9	2
Cultural values	Filial responsibility					
	Strong belief about filial responsibility	12^(1, 2, 4, 5, 6, 7, 9, 10, 13, 15, 18, 19)^	2^(22, 24)^	2^(28, 29)^	4^(34, 35, 37, 39)^	2^(45, 46)^
	Challenge of multiple roles	4^(4, 8, 10, 18)^	3^(22, 23, 24)^		2^(35, 40)^	
	Familism					
	Strong familism			1^(27)^	2^(38, 42)^	
	Respect for elderly	4^(6, 8, 17, 18)^	2^(23, 25)^		1^(35)^	
	Share of caregiving responsibility	2^(6, 15)^	1^(23)^	2^(28, 30)^		
	Conceptualizations of dementia/AD					
	Normal aging	3^(3, 16, 17)^				2^(44, 46)^
	Stigma about dementia/AD	4^(3, 17, 20, 21)^				2^(44, 46)^
	No. of articles	18	4	4	7	3
Acculturation	Challenges of acculturation					
	Traditional cultural belief	5^(4, 8, 9, 13, 15)^	3^(23, 24, 25)^	3^(27, 29, 30)^	4^(33, 34, 40, 41)^	1^(45)^
	Challenge of immigrant status	3^(9, 16, 18)^	1^(24)^		1^(37)^	1^(45)^
	Generational differences of beliefs in filial responsibility	2^(4, 9)^	2^(23, 24)^	2^(29, 31)^	2^(34, 37)^	
	No. of articles	7	3	4	5	1

*Note.* Superscript numbers refer to references given in Tables 4 to 8. Ref. No. = reference number; AD = Alzheimer's disease.

**Table 4:** Chinese American/Chinese Canadian Caregivers.

Reference	Focus of research	Research design	Sample characteristics	Key findings
(1) Chappell and Funk (2011)	Examine the differences of CC with Chinese and Caucasian CGs	Face-to-face interview	124 Chinese, 92 first- and second-generation CC, 100 Caucasian Canadian CGs	• Western culture does not have explicit norms of filial responsibility to the same extent as Chinese culture. • CC appeared more similar to homeland (Hong Kong) Chinese than host (Caucasian) Canadians.
(2) Funk, Chappell, and Liu (2011)	Examine CGs’ filial attitudes, and health and well-being	Face-to-face interview	124 Chinese, 92 first- and second-generation CC, 100 Caucasian Canadian CGs	• High filial attitudes resulted in negative health outcomes for Caucasian but protective factor for CC CGs. • CGs’ individual coping responses and social support may buffer effects of burden on self-perceived health and well-being.
(3) Gray, Jimenez, Cucciare, Tong, and Gallagher-Thompson (2009)	Examine CGs’ knowledge, attitudes, and beliefs about AD	Face-to-face interview	48 first-generation CA CGs	• CGs viewed the signs and symptoms of AD as a normal part of aging, not requiring medical interventions. • If AD as a stigmatized notion, it hindered CGs from dementia research participation and seeking professional help.
(4) Ho, Friedland, Rappolt, and Noh (2003)	Examine CGs’ feelings of caring for people with AD	Face-to-face interview	12 first-generation CC CGs	• Strong sense of obligation and anticipation of caregiving role. • Role strains and family conflicts re lack of support, family hierarchy, outside work, generational and cultural issues. • Acceptance of CG role as coping techniques.
(5) Holland, Thompson, Tzuang, and Gallagher-Thompson (2010)	Explore biopsychological response to caregiving	Face-to-face interview	47 first-generation CA CGs	• CG stressful if CR's behaviors result in shame, embarrassment, expressed negative emotions. • CGs preferred adaptive, problem-solving coping strategies. • CG's sense of filial responsibility resulted in less depression, greater self-efficacy, and positive caregiving experiences.
(6) Hsueh, Hu, and Clarke-Ekong (2008)	Explore the phenomenon of acculturation in filial practices	Focus group and face-to-face interview	21 first-generation CA CGs	• CGs shared values of collectivism and lifelong reciprocal obligations for parental care. • More educated CGs accepted mainstream values of filial care: Coordination with outside resources. • CGs with limited resources who maintain Chinese filial responsibility felt more overwhelmed.
(7) Jones, Jaceldo, Lee, Zhang, and Meleis (2001)	Examine CGs’ role involvement, role integration, and health	Questionnaire	29 first-generation CA CGs	• Role satisfaction and involvement were strongly, positively associated with CGs’ health and psychological well-being. • Little association between role integration and CGs’ health and psychological well-being was found.
(8) Jones, Zhang, Jaceldo-Siegl, and Meleis (2002)	Describe the caregiving process	Face-to-face interview	22 first-generation CA CGs	• CGs experienced being in transition between traditional Asian culture and new Western culture, beliefs, and values. • CGs conceptualized their filial commitment as “transplanted filial values.”
(9) Jones, Zhang, and Meleis (2003)	Examine parental caregiving experience	Face-to-face interview	22 first-generation CA CGs	• CGs’ determination to care at all costs increased their vulnerability due to being in transition and immigrants. • CGs connected their own inner strength with religion and grew stronger through the caregiving experience and mobilization of family resources.
(10) Lai (2007)	Examine the effects of culture on caregiving burden	Phone survey	339 first-generation CC CGs	• CGs with multiple roles had significant caregiver burden. • CGs’ shame to use external help and cultural values of respect for and duty to older adults hindered them from asking for outside support and interventions.
(11) Lai (2009a)	Understand the effect of caregiving burden on depression	Phone survey	339 first-generation CC CGs	• The higher the CGs’ burden, the more their depressive symptoms. • Health of CRs is the key determinant of burden. • The length of residency in Canada and English competency was less influential on caregiving burden.
(12) Lai (2009b)	Examine the effect of caregiving on CGs’ health	Phone survey	111 first-generation CC CGs	• Health of CRs was significantly associated with caregiving burden and related distress. • The longer the caregiving experience, the better CGs’ health because CGs adjusted to CRs’ needs, had better control over their role, and received more support, resources, or services.
(13) Lai (2010)	Examine the effect of filial responsibility on the appraisal of caregiving burden	Phone survey	339 first-generation CC CGs	• The stronger CGs’ filial responsibility, the positive their caregiving appraisals: Filial responsibility as a buffer for CGs’ psychological strengths and endurance. • Traditional Chinese cultural values and expectation (i.e., use of formal services as losing face) are pressure.
(14) Lai and Thomson (2009)	Examine the effect of social support on caregiver burden	Phone survey	340 first-generation CC CGs	• Social support was the strongest correlate of caregiving burden. • Limited financial resources, high education level, and CRs with more illnesses were associated with a high caregiving burden.
(15) Lan (2002)	Examine the cultural meaning and social practice of filial care	Face-to-face interview and observation	8 first-generation CA CRs, 8 CA CGs, and 11 home care workers	• Chinese cultural norms of filial responsibility and parental authority were modified after immigration. • The commodification of care through private or public funds became a major mechanism for immigrant families. • By recruiting paid-CGs as fictive kin, immigrant adult children maintained the cultural ideal of filial care.
(16) Levy, Hillygus, Lui, and Levkoff (2000)	Examine the quantity and types of illness that correlate with caregiver burden	Face-to-face interview	10 first-generation CA CGs	• The more attributions CGs have, the higher CGs’ burden. • Keeping traditional values and family ties was salient. • Immigration-related factors (e.g., tension and stress from living in the United States) and being an old age were salient. • Considering dementia-related cognitive and behavioral changes as normal aging led not to seek formal services.
(17) Liu, Hinton, Tran, Hinton, and Barker (2008)	Examine the relationship of dementia and stigma	Face-to-face interview	23 first-generation CA CGs	• CGs consider dementia as normal aging but with strong stigma and it brings shame and loss of face to the family. • Causes of dementia were identified as psychosocial stressors and CRs’ personal characteristics. • CRs’ status as elder is diminished due to their symptoms, but parent-child contract is preserved.
(18) Spitzer, Neufeld, Harrison, Hughes, and Stewart (2003)	Examine the experiences of CC CGs	Face-to-face interview, observation, and focus group	18 first-generation CC CGs	• CGs’ strong sense of filial responsibility, respect for elders, collectivity, and Confucian ideals were salient. • CGs believed caregiving is women's roles and rejected the idea of caregiving as a burden. • Caregiving is more costly in Canada due to smaller network and low-wage employment.
(19) Tang (2011)	Examine the positive aspects of caregiving	Face-to-face interview	113 first-generation CA CGs	• Highly acculturated CGs reported caregiving stress and burden. • CGs accepted their caregiving role as a cultural obligation. • CGs wanted their children to learn to preserve their cultural values of filial responsibility and respect for the elderly.
(20) Vickrey, Strickland, Fitten, Adams, Ortiz, and Hays (2007)	Elicit perceptions of the caregiving experience	Focus group	4 first-generation CA CGs	• CGs’ concern about CRs’ financial situation and CGs’ ability to handle future issues, lack of information about dementia and resources to assist with caregiving. • Dementia diagnosis is kept within the family due to stigma. • CGs use religion, meditation, and prayer as a source of comfort.
(21) Zhan (2004)	Examine the caregiving experiences	Face-to-face interview	4 first-generation CA CGs	• Stigma about AD brings shame to the family and isolates CRs with AD from their ethnic community. • Educational and service outreach is necessary to reduce stigmatization of AD.

*Note.* CC = Chinese Canadian; CG = caregivers; AD = Alzheimer's disease; CA = Chinese American; CR = care recipient.

**Table 5:** Filipino American Caregivers.

References	Focus of research	Research design	Sample characteristics	Key findings
(22) Jones, Jaceldo, Lee, Zhang, and Meleis (2001)	Examine CGs’ role involvement, role integration, and health	Questionnaire	21 first-generation FA CGs.	• Role integration was strongly, positively associated with CGs’ health and psychological well-being. • Role satisfaction was consistently high and significantly correlated with psychological well-being.
(23) Jones, Zhang, Jaceldo-Siegl, and Meleis (2002)	Describe the caregiving process	Face-to-face interview	19 first-generation FA CGs	• CGs experienced being in transition between traditional Asian culture and new Western culture, beliefs, and values. • CGs conceptualized caregiving as “transplanted filial values” and “high calling.” • CGs shared filial responsibilities with sons and husbands.
(24) Jones, Zhang, and Meleis (2003)	Examine parental caregiving experience	Face-to-face interview	19 first-generation FA CGs	• CGs’ strong sense of filial responsibility took priority over all other responsibilities. • CGs experienced conflicts between CGs’ and their parents’ worldviews due to acculturation. • CGs connected their inner strength with religion, grew stronger through caregiving experience, and utilized family resources.
(25) Kimura and Browne (2009)	Examine CGs’ attitudes toward caregiving and service use	Focus group and questionnaire	12 first-generation FA CGs	• CGs showed respect for the elderly and desire to reciprocate their kindness. • Immigration and economic necessity made it difficult for CGs to provide care in the United States. • CGs were receptive to formal service use and governmental assistance, but noted the issues of CRs’ shame.

*Note.* CG = caregiver; FA = Filipino American; CR = care recipient.

**Table 6:** Japanese American and Japanese Canadian Caregivers.

References	Focus of research	Research design	Sample characteristics	Key findings
(26) Anngela-Cole and Hilton (2009)	Evaluate CGs’ cultural differences in attitudes toward caregiving and stress level	Mail survey	98 third-generation JA 86 CA CGs	• Both CGs experienced similar levels of caregiving stress. • Caucasian CGs spent more caregiving time and had stronger beliefs and a more positive attitude about caregiving duties. • JA CGs used network support while Caucasian CGs relied on formal services.
(27) Knight, Robinson, Flynn Longmire, Chun, Nakao, and Kim (2002)	Assess relationship between cultural values and stress/coping	Questionnaire and face-to-face interview	20 second- and third-generation JA CGs	• CGs showed lower familism due to acculturation from Asian to Western cultural values. • Stronger Asian cultural values brought CGs higher burden. • Familism was not a buffer for this sample of CGs.
(28) Kobayashi (2000)	Explore how third-generation JA CGs support their parents	Face-to-face interview	100 second- generation JC parents and 100 third- generation JC children	• Filial responsibility was strong among the third-generation CGs. • Cultural preferences within the family, parents’ needs, children's availability, ethnic identity, and demographic factors influenced Asian American family's support.
(29)Kobayashi and Funk (2010)	Explore sense of filial responsibility across generations	Face-to-face interview	100 second- generation JC parents and 100 third- generation JC children	• Both generations regarded filial responsibility in degree and content important. • Cultural norms of filial responsibility endured in “translated,” mutually agreed ways, promoting family cohesion.
(30)Young, McCormick, and Vitaliano (2002a)	Explore attitudes toward long-term care services and utilization	Face-to-face interview	26 JA CGs, 4 CRs, and 14 professional CGs	• Ability to meet CRs’ needs, CRs’ autonomy, quality of staff, and services reflecting Japanese culture was important. • Family's involvement, sharing responsibility in overall care, communication, and coordination were important. • Bilingual JA CGs and Japanese food were preferable.
(31) Young, McCormick, and Vitaliano (2002b)	Explore CGs’ and service providers’ perspectives on community-based services	Participant observation and face-to-face interview	26 JA CGs, 4 CRs, and 14 professional CGs	• Generational differences in expectations to caregiving commitment and Japanese heritage were found. • Perceptions about caregiving became more complex and diverse as CGs became more distant from the first-generation immigrants.

*Note.* CG = caregiver; CA = Caucasian American; JA = Japanese American; JC = Japanese Canadian; CR = care recipient.

**Table 7:** Korean American Caregivers.

References	Focus of research	Research design	Sample characteristics	Key findings
(32) Casado and Sacco (2012)	Identify correlates of caregiver burden	Phone survey	146 first-generation KA CGs	• Family support rather than friends alleviated caregiver burden. • Care management efficacy positively impacted caregiver burden.
(33) Chun, Knight, and Youn (2007)	Compare caregiving distress	Face-to-face interview	63 Koreans, 53 first-generation KA, 54 CA CGs	• CRs’ behavior, memory problem, and depression affected KA CGs’ burden, depression, and anxiety. • CGs’ immigrant status exacerbated reactions to CRs’ disability due to conflicts between Korean and American values. • CGs with higher education had lower levels of anxiety.
(34) Han, Choi, Kim, Lee, and Kim (2008)	Explore caregiving experiences	Focus group	24 first-generation KA CGs	• CGs faced challenges of settling in a new country but holding on to traditional but changing value of filial responsibility. • CGs had no systematic support: Need education and culturally tailored support. • Due to different levels of acculturation within family members, conflicts arose in terms of beliefs related to caregiving.
(35) Kim and Theis (2000)	Describe the caregiving role in the KA family	Face-to-face interview	30 first-generation KA CGs	• Being CGs as “privilege,” but meant negative as no other choice but being CGs. • Caregiving while working outside home was challenging. • CGs concerned about future finances due to low-wage jobs with limited education.
(36) Kim and Knight (2008)	Investigate the effects of CG on physical health	Face-to-face interview	87 KA CGs and 87 non-CGs	• Significant associations were found between low quality of instrumental social support and CGs’ poor health (e.g., hypertension, physiological stress).
(37) Kim (2009)	Understand dementia and CGs’ postcaregiving experience	Face-to-face interview	8 first-generation KA CGs	• CGs appraised AD as a disease, accepted themselves as CGs, and caregiving as family affair. • CGs examined filial responsibility through maintaining harmony but changing its expectations across generations. • Due to immigrant status, CGs wondered if their CRs might have been in better condition if they remained in Korea.
(38) Knight, Robinson, Flynn Longmire, Chun, Nakao, and Kim (2002)	Assess the relationship between cultural values and stress/coping	Questionnaire and face-to-face interview	53 first-generation KA CGs	• Lower education, younger age, CRs’ higher depression, and embarrassment were significant predictors of higher anxiety. • KA showed higher familism and higher levels of burden and distress, indicating worse mental and physical health.
(39) Kong, Deatrick, and Evans (2010)	Describe CGs’ experiences re American nursing home placement	Face-to-face interview	10 first-generation KA CGs	• The Korean way of thinking, “family and filial piety” as a fundamental cultural belief of caregiving, was salient. • Placing a loved one to a nursing home made CGs feel inadequate. • Nursing home services were better than expected.
(40) Lee and Farran (2004)	Compare CGs’ depressive symptom	Mail survey	100 Korean, 59 first-generation KA, and 78 CA CGs	• All three groups showed high scores on depressive mode (Korean = 85%, KA = 71%, Caucasian = 63%). • KA CGs were difficult to recruit due to the conflict of filial duty and work outside home—Process of acculturation.
(41) Lee and Bronstein (2010)	Examine the role of culture in the meaning of caregiving	Mail survey	72 KA CGs	• Social support was the most important factor in determining the meaning of caregiving. • Cultural factors were insignificant in CGs’ meaning of caregiving.
(42) Yong and McCallion (2003)	Examine the meaning of caregiving experiences	Phone interview	2 first-generation KA daughters-in-law CGs	• CGs constructed meanings of their lives based on hierarchical relationships within their family. When CGs’ behavior did not fit within the relationship, it made CGs feel guilty and stressed (*hwabyung*). • Family support was the best influence on caregiving experience.

*Note.* KA = Korean American; CG = caregiver; CA = Caucasian American; CR = care recipient; AD = Alzheimer's disease.

**Table 8:** Vietnamese American Caregivers.

References	Focus of research	Research design	Sample characteristics	Key findings
(43) Hinton, Tran, Tran, and Hinton (2008)	Understand the meaning of religion/spirituality in caregiving	Face-to-face interview	9 first-generation VA CGs.	• CGs related their spirituality/religion to suffering, motivations, and understanding of dementia/ AD. • Religion and prayers helped CGs to cope with their suffering, motivate and sustain a positive attitude about caregiving.
(44) Liu, Hinton, Tran, Hinton, and Barker (2008)	Examine the relationship of dementia and stigma	Face-to-face interview	9 first-generation VA CGs	• Dementia is a normal aging process but connected with mental illness, highly stigmatized and brings shame to the family. • Traditional views of health—emphasis on the holism of minds with body and balance and harmony—were salient.
(45) Strumphf, Glicksman, Goldberg-Glen, Fox, and Logue (2001)	Explore CGs and CRs’ life experiences in a new country	Face-to-face interview	30 first-generation VA CGs and 15 first-generation VA CRs	• Acquisition of English language, limited financial resources, and assimilation to American life was a challenge. • Lack of knowledge and limited accessibility to health and social services were challenges. • Immigration, settlement, and desire to retain cultural ties and strong sense of filial responsibility impacted CGs’ lives.
(46) Yeo, Tran, Hikoyeda, and Hinton (2001)	Understand cultural conceptualization of dementia and caregiving	Face-to-face interview	9 first-generation VA CGs	• CGs strongly endorsed filial responsibility and family care. • Dementia is attributed to normal aging, physiological and psychosocial factors, and related to spiritual beliefs or fate, but social stigma is attached. • To “save face” within the community, CRs’ dementia condition was kept within the family.

*Note.* VA = Vietnamese American; CG = caregiver; AD = Alzheimer's disease; CR = care recipient.
